# *Boehmeria nivea* Stimulates Glucose Uptake by Activating Peroxisome Proliferator-Activated Receptor Gamma in C2C12 Cells and Improves Glucose Intolerance in Mice Fed a High-Fat Diet

**DOI:** 10.1155/2013/867893

**Published:** 2013-04-03

**Authors:** Sung Hee Kim, Mi Jeong Sung, Jae Ho Park, Hye Jeong Yang, Jin-Taek Hwang

**Affiliations:** Korea Food Research Institute, 516 Baekhyun-dong, Bundang-ku, Seongnam, Gyeonggi-do 463-746, Republic of Korea

## Abstract

We examined the antidiabetic property of *Boehmeria nivea* (L.) Gaud. Ethanolic extract of *Boehmeria nivea* (L.) Gaud. (EBN) increased the uptake of 2-[*N*-(nitrobenz-2-oxa-1,3-diazol-4-yl)amino]-2-deoxy-d-glucose in C2C12 myotubes. To examine the mechanisms underlying EBN-mediated increase in glucose uptake, we examined the transcriptional activity and expression of peroxisome proliferator-activated receptor gamma (PPAR-**γ**), a pivotal target for glucose metabolism in C2C12 myotubes. We found that the EBN increased both the transcriptional activity and mRNA expression levels of PPAR-**γ**. In addition, we measured phosphorylation and expression levels of other targets of glucose metabolism, such as AMP-activated protein kinase (AMPK) and protein kinase B (Akt/PKB). We found that EBN did not alter the phosphorylation or expression levels of these proteins in a time- or dose-dependent manner, which suggested that EBN stimulates glucose uptake through a PPAR-**γ**-dependent mechanism. Further, we investigated the antidiabetic property of EBN using mice fed a high-fat diet (HFD). Administration of 0.5% EBN reduced the HFD-induced increase in body weight, total cholesterol level, and fatty liver and improved the impaired fasting glucose level, blood insulin content, and glucose intolerance. These results suggest that EBN had an antidiabetic effect in cell culture and animal systems and may be useful for preventing diabetes.

## 1. Introduction

Diabetes is a serious health issue that affects the life span of humans. Of those diagnosed with diabetes, approximately 5–10% have type 1 diabetes, which is characterized by the loss of insulin production in pancreatic beta-cells, whereas 90–95% have type 2 diabetes, which is characterized by insulin resistance. Although various drugs have been developed for diabetes treatment, their side effects remain obstacles to their use. Natural ingredients are widely distributed in the plant kingdom. They have been traditionally used to treat a variety of human diseases, including metabolic disorders. It is believed that natural ingredients are safer than synthetic compounds because they have been used for a long time [1, 2]. The results of a number of studies conducted by other researchers and our previous study also suggested that naturally occurring compounds could exert beneficial health effects in the treatment of diabetes by modulating cellular signaling pathways [2–5]. Among signaling molecules, peroxisome proliferator-activated receptor gamma (PPAR-*γ*) regulates fatty acid and glucose metabolism. PPAR-*γ* has been implicated in the pathology of obesity and diabetes [3–5]. PPAR-*γ* agonists such as glitazone have been used to treat hyperglycemia [6]. In addition, PPAR-*γ* agonists derived from natural herbs may prevent diabetes. For example, *Cornus kousa* F. Buerger ex Miquel, a medicinal plant, increases PPAR-*γ* activity and stimulates glucose uptake. In addition, *Aegle marmelos* fruit aqueous extract, *Syzygium aromaticum* flower bud (clove) extract, and *Sambucus nigra* (elderflower) extract exert antidiabetic effects by increasing PPAR-*γ* activation or expression [7–9]. AMP-activated protein kinase (AMPK) and Akt protein are other important signaling molecules in glucose homeostasis. AMPK is an insulin-independent regulator of glucose uptake. By contrast, the PI3 kinase/Akt pathway is an insulin-dependent regulator of glucose uptake. Thus, AMPK and Akt are also therapeutic targets for metabolic disorders such as obesity and diabetes [10, 11].

*Boehmeria nivea* (L.) Gaud., a flowering plant in the nettle family Urticaceae, has been widely grown in east Asian countries such as Korea, India, and China. The edible parts of this plant, the leaves and roots, have been reported to have anti-inflammatory, antioxidant, and antifungal effects [12, 13]. However, the antidiabetic effect of *B. nivea* has not been clearly elucidated. Therefore, in this study we evaluated the antidiabetic potential of ethanol extract of *B. nivea* (EBN) and its signaling mechanisms by using *in vitro* and *in vivo* approaches.

## 2. Materials and Methods

### 2.1. Materials

An authenticated *B. nivea* sample was provided by a public officer from the Seocheon County Office (Seocheon, Republic of Korea), where a voucher specimen has been deposited. C2C12 and HEK293 cells were purchased from the American Type Culture Collection (Manassas, VA, USA). Dulbecco's modified Eagle's medium (DMEM) and fetal bovine serum (FBS) were purchased from Welgene (Daegu, Republic of Korea). Horse serum was purchased from Life Technologies (Seoul, Republic of Korea). 2-[*N*-(Nitrobenz-2-oxa-1,3-diazol-4-yl)amino]-2-deoxy-d-glucose (2-NBDG) was purchased from Invitrogen (Carlsbad, CA, USA). PPAR-*γ* and glyceraldehyde phosphate dehydrogenase (GAPDH) primers were designed based on sequence data from the NCBI database and were purchased from Bioneer (Daejeon, Republic of Korea). Phospho-AMP-activated protein kinase (pAMPK), phospho-Akt (pAKT), PPAR-*γ*, and AMPK were purchased from Cell Signaling Technology (Beverly, MA, USA). *β*-actin and horseradish peroxidase-conjugated secondary antibody were purchased from Santa Cruz Biotechnology (Santa Cruz, CA, USA). 

### 2.2. Extraction and Lyophilization

High-quality *B. nivea* specimens were selected and mixed by a specialist, and the ethanol extract was prepared. Leaves of *B. nivea* were pulverized and extracted with 70% ethanol by shaking for 24 h at 25°C, and the precipitates were removed by centrifugation at 8,000 ×g for 30 min (Beckman, USA). Supernatants were lyophilized using a freeze dryer (Il Shin, Dongducheon, Republic of Korea). The yield of ethanol extract from the leaves of *B. nivea* was 10.0% (w/w). Ethanol extract from the leaves of *B. nivea* (EBN) was dissolved in distilled water and sterilized by passage through a 0.45 *μ*m Millipore filter unit for use in the experiments. 

### 2.3. Muscle Cell Differentiation and Glucose Uptake Assay

C2C12 cells were cultured in DMEM containing 10% FBS. The cells were maintained at 37°C in a humidified 5% CO_2_ environment. After the cells reached confluency, the medium was changed to DMEM supplemented with 2% horse serum, until the cells were entirely differentiated. For the experiments, the cells were starved in low-glucose serum-free DMEM for 12 h and then treated with or without 50 *μ*M 2-NBDG or with 2-NBDG with EBN at the indicated concentrations (200, 400, 800, and 1200 *μ*g/mL) for 24 h. Cellular uptake of 2-NBDG was measured using a fluorometer at excitation and emission wavelengths of 465 and 540 nm, respectively.

### 2.4. PPAR-*γ* Transcriptional Activity Assay

PPAR-*γ* transcriptional activity was measured as described previously [14]. HEK293 cells were cultured in DMEM containing 10% FBS. Cells were transiently transfected with 1 *μ*g of total DNA (expression plasmids for PPAR-*γ*, retinoid X receptor *α* (RXR*α*), PPAR response elements (PPREs), and *β*-galactosidase) by using SuperFect Transfection Reagent (Qiagen, Valencia, CA, USA) according to the manufacturer's instructions. After transfection, the cells were treated with EBN at the indicated concentrations (200, 400, 800, and 1200 *μ*g/mL) in the absence or presence of rosiglitazone, a PPAR-*γ* agonist, for 24 h. PPAR-*γ* transcriptional activity was examined using a luciferase reporter gene assay with the Luciferase Assay System (Promega, Madison, WI, USA) and was normalized to the *β*-galactosidase activity. 

### 2.5. RNA Isolation and Reverse Transcriptase-Polymerase Chain Reaction

Differentiated C2C12 cells were treated with various concentrations of EBN for 6 h, and total RNA was isolated using TRIzol reagent (Invitrogen) according to the manufacturer's instructions. cDNA was synthesized from isolated RNA and was amplified by polymerase chain reaction (PCR) in a PCR thermal cycle device by using specific primers: PPAR-*γ* (sense), 5′-ACC ACT CGC ATT CCT TTF AC-3′; PPAR-*γ* (antisense), 5′-TCA GCG GGA AGG ACT TTA TG-3′; *β*-actin (sense), 5′-TCA CCC ACA CTG TGC CCA TCT ACG A-3′; and *β*-actin (antisense), 5′-GGA TGC CAC AGG ATT CCA TAC CCA-3′.

### 2.6. Protein Extraction and Western Blot Analysis

Total protein was extracted from EBN-stimulated cells by using lysis buffer (50 mM Tris-HCl, pH 8.0, 5 mM EDTA, 150 mM NaCl, 1% NP-40, 1 mM PMSF, a protease-inhibitor cocktail, and a phosphatase-inhibitor cocktail). Equal amounts of protein (30 *μ*g) were separated using 10% SDS-PAGE and were transferred onto a nitrocellulose membrane. The membrane was blocked with 5% skim milk in Tris-buffered saline (TBS) for 1 h and was incubated in primary antibody diluted in TBS. After washing with TBST (TBS with 0.1% Tween 20), the membrane was incubated with HRP-conjugated secondary antibody for 1 h at room temperature. Blots were developed with an enhanced chemiluminescence (ECL) kit (Amersham, Buckinghamshire, UK).

### 2.7. Animal Experiments

Male C57BL/6J mice (age, 3 weeks) were obtained from Nara Biotech (Seoul, Republic of Korea) and housed under a 12 h light/12 h dark cycle in a temperature- and humidity-controlled room (24°C ± 1°C at 50% relative humidity). After adaptation for 1 week, the mice were freely fed a 10% fat normal diet (ND, D12450B, Research Diets, New Brunswick, NJ, USA), a 60% kcal high-fat diet (HFD, D12492, Research Diets, New Brunswick, NJ, USA), or a 60% kcal high-fat diet plus 0.5% EBN (HFD + 0.5% EBN) for 9 weeks. Food intake and body weight were measured every week. After 9 weeks, the mice were fasted overnight and then killed. The blood samples were collected from the orbital vein. All animal experiments were approved by the Institutional Animal Care and Use Committee of the Korea Food Research Institute.

### 2.8. Glucose Tolerance Test

The glucose tolerance test (GTT) was performed at 8 weeks. After overnight fasting, mice were intraperitoneally administered glucose (1 g/kg of body weight), and blood was collected from the tail vein every 30 min from 0 min to 150 min after injection. Blood glucose levels were examined by an Accu-Chek glucometer (Roche, Basel, Switzerland).

### 2.9. Quantitation of Serum Total Cholesterol and Insulin Levels

Fasting serum levels of total cholesterol (TC) and insulin were determined by enzymatic methods using commercial kits (Asan Pharm, Seoul, Republic of Korea).

### 2.10. Histopathology

Liver tissue was fixed in 4% buffered formalin and cut into 4 *μ*m thick sections. The sections were stained with hematoxylin and eosin (H&E) and examined by microscopy. Fat content was scored semiquantitatively with the following parameters, as described previously [15]: 0 = no fat; 1+ = <25%, 2+ = 25–50%, 3+ = 51–75%, 4+ = 76–95%, and 5+ = 100%.

### 2.11. Cytotoxicity Test

Cells were starved with low-glucose, serum-free DMEM for 12 h and treated with EBN at the indicated concentrations (200, 400, 800, and 1200 *μ*g/mL) for 24 h. The medium was removed, and the cells were incubated with 100 *μ*L 3-(4,5-dimethylthiazol-2-yl)-2,5-diphenyltetrazolium bromide (MTT) solution (Sigma-Aldrich, St. Louis, MO, USA) in PBS at 5 mg/mL for 4 h. The absorbance was measured using an ELISA reader at 540 nm.

### 2.12. Statistical Analysis

The data have been expressed as the mean ± standard deviation (SD) values of at least 3 independent experiments. Statistical analyses were performed using SPSS version 9.0 (SPSS Inc., Chicago, IL, USA). Differences between means were evaluated using two-way analysis of variance (ANOVA) followed by the Bonferroni test. Values of *P* < 0.05 were considered significant.

## 3. Results

### 3.1. EBN Stimulates Glucose Uptake in C2C12 Myotubes

**Figure 1:**
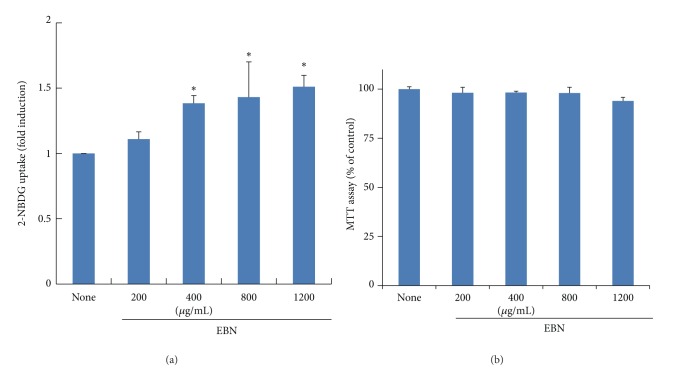
Effect of EBN on glucose uptake in C2C12 myotubes. Differentiated C2C12 cells were treated with EBN and 2-[*N*-(nitrobenz-2-oxa-1,3-diazol-4-yl) amino]-2-deoxy-d-glucose (2-NBDG) for 24 h. Then, the 2-NBDG assay was performed as described in Section 2 (a). After treatment of EBN for 24 h in C2C12 myotubes, the cytotoxicity was measured by MTT assay as described in Section 2. The result of MTT assay was expressed as percentage of control (None) (b). Data are expressed as mean ± standard deviation (SD); *n* = 3. *P < 0.05 versus None.

We first performed the 2-NBDG uptake assay to examine the antidiabetic activity of EBN in C2C12 myotubes. EBN significantly increased 2-NBDG uptake in a dose-dependent manner (Figure 1(a)). Cytotoxic effects of EBN were not observed below 1200 *μ*g/mL (Figure 1(b)). This result suggests that EBN increases glucose uptake in myotubes.

### 3.2. Activation of PPAR-*γ*, rather than AMPK and Akt, Is Involved in EBN-Stimulated Glucose Uptake

**Figure 2:**
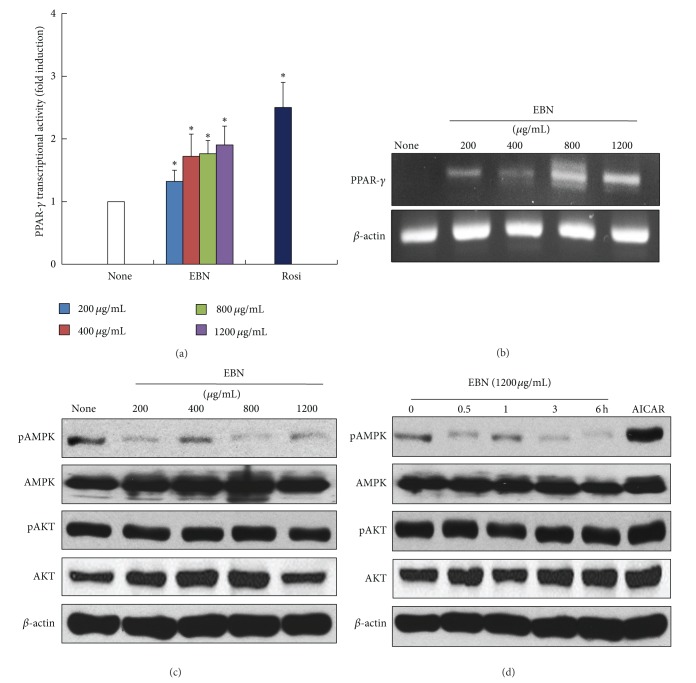
The effect of EBN on transcriptional activity and expression of peroxisome proliferator-activated receptor gamma. HEK293 cells were transiently transfected with luciferase construct containing peroxisome proliferator-activated receptor gamma (PPAR-*γ*), retinoid X receptor (RXR*α*), PPAR-response element (PPRE), and *β*-galactosidase. Then, the cells were treated with ethanol extract of *Boehmeria nivea* (EBN) (200, 400, 800, and 1200 *μ*g/mL) for 24 h. Luciferase assay was performed, and the activity was normalized with that of *β*-galactosidase activity (a). The differentiated C2C12 cells were exposed to EBN in a dose-dependent manner for 6 h. The mRNA level of PPAR-*γ* and GAPDH was measured by reverse transcriptase-polymerase chain reaction (RT-PCR) (b). Differentiated C2C12 cells were treated withEBN in a dose- and time-dependent manner. AMP-activated protein kinase (AMPK) and Akt expressions were determined by western blot analysis ((c), (d)). 1 mM AICAR was used as positive control for AMPK activation. Data are expressed as mean ± standard deviation (SD); *n* = 3. Statistical significance: *P < 0.05 for the none versus EBN or rosiglitazone.

To determine the molecular mechanisms underlying EBN-stimulated glucose uptake, we measured the activities of PPAR-*γ*, AMPK, and Akt. PPAR-*γ* is a critical target of a number of insulin-sensitizing drugs [3–5]. Treatment with EBN significantly increased the transcriptional activity of PPAR-*γ* in HEK293 cells (Figure 2(a)). Under the same conditions, 25 *μ*M rosiglitazone was used as a positive control for PPAR-*γ* activation. In addition, we measured the expression of PPAR-*γ* in EBN-treated C2C12 myotubes. EBN increased the expression of PPAR-*γ* in C2C12 myotubes (Figure 2(b)). Then, we examined whether AMPK and Akt signaling pathways were involved in EBN-stimulated glucose uptake. AMPK is another target for the antidiabetic effect of metformin, and Akt is a critical mediator of the insulin-sensitizing effect [10, 11]. EBN did not increase the phosphorylation or expression of AMPK and Akt in a dose- and time-dependent manner in C2C12 myotubes (Figures 2(c) and 2(d)). *5-*Aminoimidazole-4-carboxamide 1-*β*-D-ribofuranoside (AICAR) was used as a positive control for AMPK activation. These results indicate that EBN stimulates glucose uptake, at least in part through activation of PPAR-*γ*. AMPK and Akt signaling pathways, however, were not involved in EBN-stimulated glucose uptake in C2C12 myotubes.

### 3.3. Administration of EBN Improves Body Weight, Body Fat Mass, Liver Fat Content, and Serum TC Levels in Mice Fed a HFD

**Figure 3:**
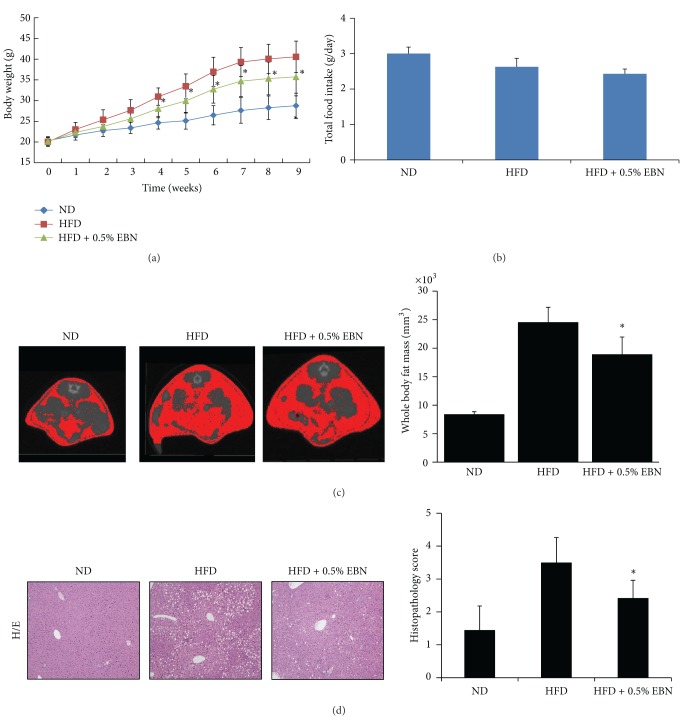
The effect of EBN on body weight, total food intake, and accumulation of fat in the liver of mice fed a high-fat diet. Mice were fed normal diet (ND), high-fat diet (HFD), and high-fat diet with 0.5% EBN (HFD + 0.5% EBN). Body weight and food intake were measured every week ((a), (b)). Body fat mass was assessed using computed tomography (CT) during the 8 weeks (c). In addition, we measured the accumulation of fat in the liver as described in Section 2 (d). Data are expressed as the mean ± standard deviation (SD) (*n* = 10). Statistical significance: *P < 0.05 for the HFD versus HFD + 0.5% EBN.

We performed an *in vivo* experiment to confirm the antidiabetic effect of EBN. The initial body weights of mice in each group were not statistically different (Table 1). The final body weights were lower in the EBN group (Table 1). The body weights in the HFD group were higher than those in the ND group (Figure 3(a)). The body weights of mice in the HFD + 0.5% EBN group were lower than those in the HFD group. The total food intake was not statistically different in each group (Figure 3(b)). In addition, we assessed body fat mass by using computed tomography (CT) imaging and found that the whole body fat mass was higher in the HFD group than in the ND group, which is shown in red in Figure 3(c). Administration of 0.5% EBN significantly reduced the whole body fat mass in mice fed a HFD (Figure 3(c)). Further, serum cholesterol levels were significantly lower in the HFD + 0.5% EBN group than in the HFD group (Table 1). Moreover, the concentration of fat in the liver tissue was significantly lower in the HFD + 0.5% EBN group than in the HFD group. The histopathology scores also showed that 0.5% EBN significantly decreased fat accumulation stimulated by HFD (Figure 3(d)).

### 3.4. Effect of EBN on Fasting Glucose Levels and Glucose Intolerance

**Figure 4:**
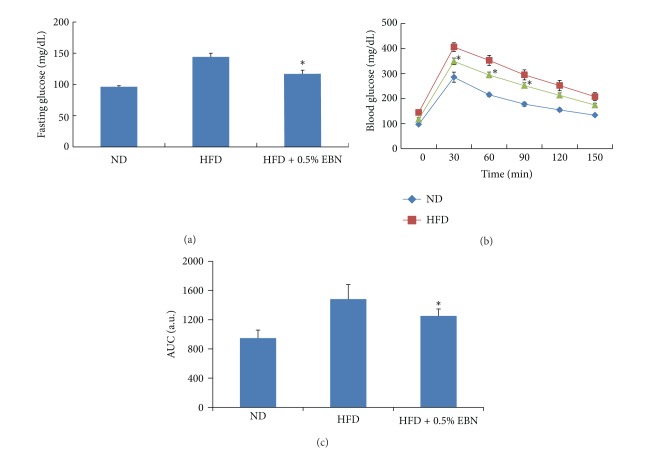
The effect of EBN on fasting glucose levels and glucose intolerance in mice fed a high-fat diet. Mice were starved for 12 h and blood was drawn from the orbital vein, and we measured the fasting glucose levels as described in Section 2 (a). Glucose tolerance test was performed at the end of 8 weeks. Mice were injected with glucose (1 g/kg of body weight), and blood glucose levels were examined every 30 min from 0 min to 150 min after injection (b). Area under curve (AUC) during glucose tolerance test (c). Data are expressed as mean ± standard deviation (SD) (*n* = 10). Statistical significance: *P < 0.05 for the high-fat diet (HFD) versus HFD plus 0.5% EBN.

We determined fasting glucose and insulin levels and performed a GTT to determine the antidiabetic effect of EBN in mice fed a HFD. The fasting glucose level was significantly higher in the HFD group than in the ND group. The fasting glucose level was lower in the HFD + 0.5% EBN group than in the HFD group (Figure 4(a) and Table 1). Compared to the ND group, the HFD group showed an abnormal increase in insulin levels, but the insulin levels significantly decreased in the HFD + 0.5% EBN group (Table 1). The GTT test showed that the HFD led to glucose intolerance. Administration of 0.5% EBN markedly decreased the glucose intolerance induced by the HFD (Figures 4(b) and 4(c)). These results show that EBN improves glucose tolerance and insulin resistance. 

## 4. Discussion

In this study, we showed that EBN exerts an antidiabetic effect in C2C12 myotubes and in a mouse model of a HFD. In addition, we showed that PPAR-*γ* activation, rather than AMPK and Akt signaling, was associated with the antidiabetic activity of EBN. Our findings demonstrate that EBN has antidiabetic and antiobesity effects *in vitro* and *in vivo*. Compared to the findings of a recent study that showed that *B. nivea* inhibits alpha-glucosidase or beta-glucosidase, our findings better substantiate the antidiabetic effect of EBN [16]. Although the recent study showed the glucosidase inhibitory effect of *B. nivea in vitro*, it did not describe the mechanisms underlying this effect; our study is based on these data, and we performed experiments to confirm the antidiabetic effects of *B. nivea.* Through cell and animal experiments, we demonstrated the precise antidiabetic efficacy of *B. nivea* and the mechanisms underlying the effect. The total phenolic content of EBN was 3640 mg/100 g. Several phenolic compounds such as rutin (46.48 mg/100 g), chlorogenic acid (1.96 mg/100 g), luteolin-7-glucoside (11.29 mg/100 g), naringin (1.13 mg/100 g), hesperidin (23.69 mg/100 g), and tangeretin (1.59 mg/100 g) have already been found in EBN [17]. 

We showed that EBN-stimulated glucose uptake is accompanied by PPAR-*γ* activation and expression but is not associated with AMPK and Akt activation. PPAR-*γ* participates in glucose uptake, and PPAR-*γ* agonists increase the sensitivity of insulin receptors [3–5]. In addition to thiazolidinedione, a PPAR-*γ* agonist that is used clinically, naturally occurring ingredients or compounds have also been used as PPAR-*γ* agonists to significantly improve insulin resistance [3, 18, 19]. For example, *Cornus kousa* F. Buerger ex Miquel, a medicinal plant, increases PPAR-*γ* ligand binding activity and stimulates glucose uptake by an AMPK-independent mechanism [7]. A number of studies have shown that aqueous extract from the fruits of *Aegle marmelos*, *Syzygium aromaticum* flower bud (clove) extract, and *Sambucus nigra* (elderflower) extract improve insulin resistance by increasing PPAR-*γ* activation or expression [8, 9]. Therefore, consistent with previous studies, PPAR-*γ* activation and expression could explain the antidiabetic effect of EBN observed in our study. AMPK and Akt were not altered by EBN under our experimental conditions. AMPK is a key regulator of glucose metabolism and is a target of metformin, an antidiabetic drug [10]. Like PPAR-*γ*, AMPK is a target for naturally occurring ingredients or compounds for the prevention of diabetes [20]. Insulin-dependent PI3K/Akt signaling can also improve diabetes by activating insulin receptor [21]. Once insulin binds to insulin receptor, insulin receptor substrate 1 (IRS-1) is activated. Activated IRS-1 triggers insulin signaling cascades involving PI3K, phosphoinositide-dependent kinase-1 (PDK-1), and Akt and increases glucose transporter 4 (Glut-4) translocation and glucose uptake [22]. EBN treatment did not alter insulin-dependent PI3K/Akt signaling and AMPK signaling (Figures 2(c) and 2(d)). These results indicate that EBN-stimulated glucose uptake is mediated by PPAR-*γ*, which suggests that EBN does not mimic the effect of insulin through the insulin receptor or the effect of metformin on AMPK activation. To our knowledge, this is the first study to show the antidiabetic effect and mechanisms of EBN. However, it is still unclear how EBN acts as a PPAR-*γ* agonist. One possibility, hinted at by early reports, is that PPAR-*γ* agonists stimulate glucose uptake by increasing Glut4 expression and mRNA levels [23]. In addition, elevated levels of PPAR-*γ* transcript are accompanied by enhanced Glut-4 transcription and glucose uptake, suggesting that glucose uptake and Glut-4 transcription are induced through the activation of PPAR-*γ* by PPAR-*γ* agonists [24]. Therefore, the antidiabetic effect of EBN may be due to increased PPAR-*γ* and Glut-4 transcription. However, further studies are required to explain the cascades downstream from EBN-mediated activation of PPAR-*γ* that stimulate glucose uptake. 

Administration of 0.5% EBN significantly reduced body weight, body fat mass, liver fat content, and serum total cholesterol levels. In addition, EBN significantly improved HFD-induced fasting glucose levels and glucose tolerance. A number of studies have described a link between HFD-induced obesity and metabolic disorders, including diabetes, hyperlipidemia, and hypercholesterolemia [25, 26]. Obesity causes insulin resistance and thus results in type 2 diabetes. We have shown that a HFD accelerated obesity and glucose intolerance. Administration of EBN dramatically improved the glucose intolerance caused by a HFD. Sancheti et al. showed that the root extract of *B. nivea* exerts an antidiabetic effect against streptozotocin- (STZ-) induced diabetes in rats [16]. In fact, the STZ-induced diabetic model is generally used to identify antidiabetic drugs or ingredients that offset type 1 diabetes. In the present study, we investigated the antidiabetic effect of the leaf extract of *B. nivea*. We used the leaf extract because the leaves of* B. nivea* have traditionally been ingested and used for the prevention of certain diseases in Oriental countries. The results reported by Sancheti et al. on the antidiabetic effect of the root extract of *B. nivea* in a type 1 diabetes model indicated that *B. nivea* may be effective for the prevention of metabolic disorders such as diabetes, obesity, and fatty liver [27]. As expected, we found that EBN was effective in obesity-induced type 2 diabetes mice. Taken together, these findings indicate that the preventative effects of EBN are applicable to obesity-induced insulin resistance similar to that in type 2 diabetes. In addition, the serum levels of total cholesterol and the fat content of the liver decreased in the EBN-treated group. An elevated total cholesterol level is a risk factor for obesity and cardiovascular diseases. Previous papers have also reported that a number of ingredients extracted from plants exert a cholesterol-lowering effect by lowering the absorption of cholesterol and increasing fecal sterol excretion [28, 29]. In addition, several compounds found in plants inhibit cholesterol synthesis and reduce low-density lipoprotein- (LDL-) cholesterol levels in the plasma [30, 31]. Sancheti et al. have also reported that* B. nivea* exerts an inhibitory effect on cholinesterase *in vitro*, suggesting that *B. nivea* has the potential to lower cholesterol levels [16]. Cholinesterase plays an important role in the metabolism of lipids. Elevation of the serum cholinesterase level is accompanied by high serum cholesterol levels [16]. Thus, the cholesterol-lowering effect of EBN may be due to increased cholesterol excretion or decreased cholesterol synthesis in the liver. The cholesterol lowering effect of EBN may also be due to reduced serum levels of cholinesterase. However, the mechanisms by which EBN lowers serum cholesterol levels should be elucidated further in future studies. 

## 5. Conclusions

Our study showed that EBN exerted an antidiabetic effect by targeting PPAR-*γ* signaling in myotubes. In addition, EBN improved the abnormal increase in body weight, liver fat, and serum cholesterol level observed in HFD-fed mice. Therefore, *B. nivea* may be useful for preventing diabetes. However, additional studies are required to explain the precise signaling cascades underlying the antidiabetic effect of *B. nivea* in both cell and animal models. 

## Figures and Tables

**Table 1:** Effect of EBN on high-fat diet-induced body weight changes, serum cholesterol, and insulin.

	ND	HFD	EBN
Initial body weight (g)	20.3 ± 0.9	20.0 ± 1.1	20.1 ± 0.9
Final body weight (g)	28.8 ± 3.0	40.6 ± 3.8^a^	35.7±4.6^b^
Serum			
Total cholesterol (mg/dL)	172 ± 14	209 ± 9^a^	196 ± 9^b^
Insulin (ng/mL)	0.331 ± 0.112	1.22 ± 0.21^a^	0.812 ± 0.201^b^

ND: normal diet; HFD: high-fat diet; EBN: high-fat diet plus 0.5% EBN. Data are expressed as mean ± SD (*n* = 10). Statistical significance: ^a^ *P *< 0.05 for the ND versus HFD; ^b^ *P* < 0.05 for the HFD versus HFD + 0.5% EBN.
